# Quantitative Phosphoproteomic and System-Level Analysis of TOR Inhibition Unravel Distinct Organellar Acclimation in *Chlamydomonas reinhardtii*

**DOI:** 10.3389/fpls.2018.01590

**Published:** 2018-11-28

**Authors:** Valentin Roustan, Wolfram Weckwerth

**Affiliations:** ^1^Department of Ecogenomics and Systems Biology, University of Vienna, Vienna, Austria; ^2^Vienna Metabolomics Center (VIME), University of Vienna, Vienna, Austria

**Keywords:** TOR, rapamycin, proteomics, phosphoproteomics, energy signaling, plant systems biology, biofuels

## Abstract

Rapamycin is an inhibitor of the evolutionary conserved Target of Rapamycin (TOR) kinase which promotes and coordinates translation with cell growth and division. In heterotrophic organisms, TOR regulation is based on intra- and extracellular stimuli such as amino acids level and insulin perception. However, how plant TOR pathways have evolved to integrate plastid endosymbiosis is a remaining question. Despite the close association of the TOR signaling with the coordination between protein turn-over and growth, proteome and phosphoproteome acclimation to a rapamycin treatment have not yet been thoroughly investigated in *Chlamydomonas reinhardtii*. In this study, we have used *in vivo* label-free phospho-proteomic analysis to profile both protein and phosphorylation changes at 0, 24, and 48 h in *Chlamydomonas* cells treated with rapamycin. Using multivariate statistics we highlight the impact of TOR inhibition on both the proteome and the phosphoproteome. Two-way ANOVA distinguished differential levels of proteins and phosphoproteins in response either to culture duration and rapamycin treatment or combined effects. Finally, protein–protein interaction networks and functional enrichment analysis underlined the relation between plastid and mitochondrial metabolism. Prominent changes of proteins involved in sulfur, cysteine, and methionine as well as nucleotide metabolism on the one hand, and changes in the TCA cycle on the other highlight the interplay of chloroplast and mitochondria metabolism. Furthermore, TOR inhibition revealed changes in the endomembrane trafficking system. Phosphoproteomics data, on the other hand, highlighted specific differentially regulated phosphorylation sites for calcium-regulated protein kinases as well as ATG7, S6K, and PP2C. To conclude we provide a first combined *Chlamydomonas* proteomics and phosphoproteomics dataset in response to TOR inhibition, which will support further investigations.

## Introduction

Living organisms need to continuously adjust their cell growth to their energy status. All known organisms store energy in form of ATP via nutrient metabolization. Therefore, it is crucial to sense the nutritional input to coordinate the organisms growth. In yeast, nutrient availability is sensed by the evolutionary conserved Target Of Rapamycin complex (TORC) (Dobrenel et al., 2016a; Roustan et al., 2016). TORC, as well as its signaling pathway, is conserved in most of the lower and higher eukaryotes present in the three eukaryotic kingdoms (van Dam et al., 2011; Laplante and Sabatini, 2012; Xiong and Sheen, 2015; Couso et al., 2016; Roustan et al., 2016). While in human and yeast the TOR kinase can form two different complexes (TORC1 and TORC2), only TORC1 is conserved in plants and algae like *Chlamydomonas* (van Dam et al., 2011; Roustan et al., 2016). TOR associated proteins; LST8 and RAPTOR have been identified in *Chlamydomonas* and are conserved along the viridiplantae lineage (Crespo et al., 2005; Díaz-Troya et al., 2008a,b; Shemi et al., 2015). It was found that rapamycin was able to inhibit TOR activity in yeast and mammals and *Chlamydomonas* but not in Arabidopsis (Heitman et al., 1991; Vézina and Kudelski, 1975; Xu et al., 1998; Crespo et al., 2005; Sormani et al., 2007; Xiong and Sheen, 2012; Aylett et al., 2016). Mechanistically, TOR is inhibited by the complex formed between FKBP12 (a prolyl-*cis,trans*-isomerase protein) and rapamycin which in-turn impairs the TOR kinase domain and its targeted substrates. Consequently, a rapamycin treatment in *Chlamydomonas* induces a growth inhibition as well as a vacuolarization of the cell (Crespo et al., 2005). In plants, recent publications have highlighted the potential relationship between nutrient status and TOR activation. More particularly, those studies have studied the connection between TOR signaling, nutrient deprivation (such as nitrogen depletion), sulfur and inositol metabolisms (Pérez-Pérez and Crespo, 2010; Perez-Perez et al., 2010; Imamura et al., 2015, 2016; Couso et al., 2016; Dong et al., 2017; Roustan et al., 2017). While the knowledge about TOR signaling in yeast and mammals has a solid background the related signaling networks in plants and in particular in *Chlamydomonas* are rather underexplored. Nevertheless, some components regulated by TORC1 complex in *Chlamydomonas* have been highlighted with the use of rapamycin treatment. Indeed, it appears that TOR is involved in the control of protein synthesis and especially in the activity of the endoplasmic reticulum (ER) (Diaz-Troya et al., 2011). However, unlike in higher plants, yeast, and mammals, no direct link between TOR activation and the promotion of rRNA translation via the phosphorylation cascade involving the S6 Kinase protein and the ribosomal protein S6 were found in *Chlamydomonas* (Chung et al., 1992; De Virgilio and Loewith, 2006; Ma and Blenis, 2009; Schepetilnikov et al., 2011; Xiong and Sheen, 2012; Dobrenel et al., 2016b). While TOR is activating the protein synthesis under nutrient availability, several studies have pointed out the negative regulation of autophagy processes by TOR signaling in *Chlamydomonas*. Conserved autophagy-related genes (ATG) have been found in photosynthetic organisms and molecular data support the activation of autophagy during rapamycin treatment and nutrient deprivation via the ATG8 accumulation to the autophagosome in *Chlamydomonas* (Díaz-Troya et al., 2008b; Pérez-Pérez and Crespo, 2010; Perez-Perez et al., 2010; Perez-Perez and Crespo, 2014; Shemi et al., 2015). Additionally, several studies have analyzed the metabolic acclimation of *Chlamydomonas* to rapamycin treatment which also reveals similarity to nitrogen stress adaptation. A striking example, reported by several authors, indicate a tri-acyl-glycerol (TAG) accumulation during rapamycin treatment in green algae, including *Chlamydomonas* (Imamura et al., 2015; Couso et al., 2016; Mukaida et al., 2016) but also in distant red algae (Imamura et al., 2016) during nitrogen depletion (Breuer et al., 2013; Goodenough et al., 2014; Yang et al., 2015). TAG accumulation is of a major interest since it can be used for lipid and biodiesel production (Merchant et al., 2012; Liu and Benning, 2013). More dramatically, rapamycin treatment affects directly the central primary metabolism. Further, metabolite profiling unraveled important changes for intermediates of the TCA cycle and amino acid metabolism, especially for methionine and cysteine metabolism deeply interconnected with the redox homeostasis (Foyer and Noctor, 2011; Ren et al., 2012; Caldana et al., 2013; Lee and Fiehn, 2013; Kleessen et al., 2015; Juppner et al., 2017). Finally, transcriptomic analysis has shed light on the role of TOR on the transcriptome regulation and its coordination with the metabolome (Kleessen et al., 2015). Indeed, *Chlamydomonas* transcriptomic analysis has shown that TOR inhibition up-regulated genes involved in tetrapyrrole synthesis, vacuolar function, amino acid metabolism and transport as well as folding and chaperonin related genes. On the other hand, genes involved in nucleotide metabolism, cell cycle and DNA replication and repair were down-regulated.

Considering these results, we conducted a proteomics and phosphoproteomics survey to better understand the molecular response of *Chlamydomonas* to rapamycin-induced TOR inhibition. We analyzed the influence of rapamycin treatment on cell physiology, proteome, and phosphoproteome using a cell wall deficient strain CC-503 during a 0, 24, and 48 h TOR time course under mixotrophic growth conditions and continuous light. The results were compared to transcriptomics and metabolomics studies. Rapamycin treatment induced cell growth inhibition, starch, and TAG accumulation. Proteomics and phosphoproteomics data were subjected to statistical analysis and functional annotation to provide a system-level investigation of stress perception and transduction. Significant changes in both protein and phosphorylation pattern were further investigated with the help of STRING/protein–protein interaction networks (von Mering et al., 2005). Altogether, our data shed light on the plastidial interconnection between nucleotide synthesis, sulfur, serine, methionine and cysteine metabolism. As well, our data suggest an uncoupling between plastid and mitochondria metabolism in response to translation inhibition. Eventually, *in vivo* quantitative phosphoproteomics data highlight direct consequences of TOR signaling with specific changes of phosphorylations sites in TOR targets S6K and ATG7 proteins as well as the upstream regulators such as PP2C.

## Results

### Characterization of *Chlamydomonas reinhardtii* Growth and Physiological Parameter During Rapamycin Treatment

**FIGURE 1:**
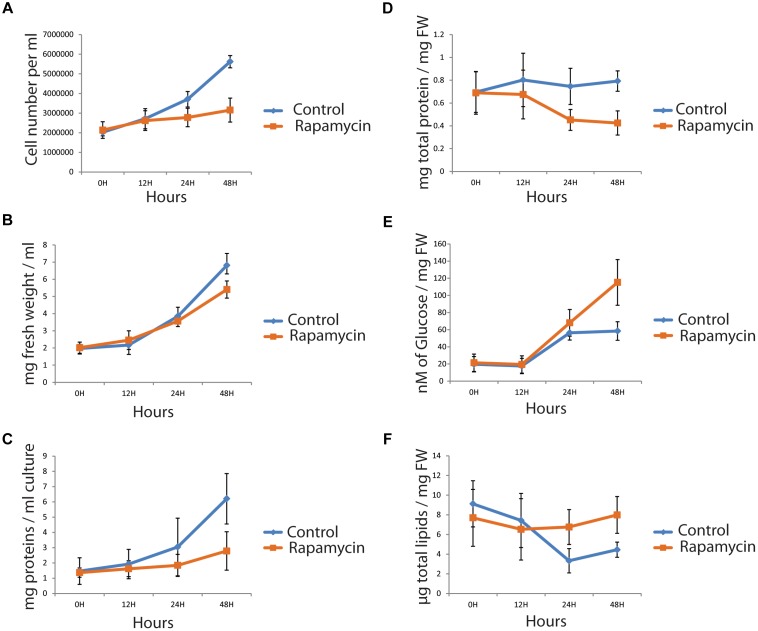
Phenotyping of *Chlamydomonas reinhardtii* cells, treated with 500 nM rapamycin (orange) or with drug vehicle (blue). A time course (0, 24, and 48 h) was sampled and cell number **(A)**, fresh weight **(B)** and total protein per ml **(C)** were measured. Further, protein **(D)**, starch (in μM of glucose/mg FW) **(E)** and total lipid content **(F)** were measured and normalized by the fresh weight. (*n* = 4, *p* < 0.05).

Support for a specific TOR regulation under nitrogen depletion and recovery was recently published (Roustan et al., 2017). To allow data comparison, we applied similar growth condition in the present study (see Materials and Methods). Additionally, cells were treated with 500 nM rapamycin or drug vehicle and sampled at 0, 24, and 48 h to measure multiple physiological parameters. Remnant growth was still observed using both cell number and fresh weight (FW) level in rapamycin-treated samples (Figures 1A,B). Indeed *Chlamydomonas* growth is not fully arrested by rapamycin treatment even with 10 μM rapamycin as previously reported (Crespo et al., 2005; Juppner et al., 2017). Protein content per milliliter was assessed with Bradford assay (see Materials and Methods). Measured protein concentration per milligram fresh weight decreased by 40% (Figures 1C,D). Protein content result is in line with the previously measured [^14^C]Arg incorporation in *de novo* protein synthesis (Diaz-Troya et al., 2011). Furthermore, photosynthetic activity was measured as well as chlorophyll content. In both cases, PSII efficiency (Fv/Fm) and total chlorophyll content per milligram fresh weight, presented no significant difference between control and rapamycin-treated samples, indicating that photosynthetic activity is not altered by TOR inhibition (Supplementary Figure S1). In this context, maintenance of photosynthetic activity while protein synthesis decrease should induce an accumulation of carbon storage compounds. Several studies have correlated cell growth inhibition to carbon storage molecules accumulation like starch and lipids (Merchant et al., 2012; Juergens et al., 2016). To decipher how the carbon pool is preferentially stored, starch and lipids were extracted as described in materials and methods; starch content was determined by an enzymatic assay and total lipid content was weighted (see Materials and Methods). Unlike in nitrogen depletion, lipid content was found to be stable during rapamycin treatment (Figure 1F). However, the starch content increased up to 50% at 48 h, compared to control samples (Figures 1E,F). Complementary, we investigated whether some lipid classes, like Tri-Acyl-Glycerol (TAG), could accumulate during TOR inhibition, as in nitrogen depletion. To investigate this possibility, time-distribution of lipid class was analyzed by thin-layer-chromatography (Fuchs et al., 2011). We found that TAG accumulates during TOR inhibition (Supplementary Figures S1D,E). Taken together, our results indicate, that unlike nitrogen stress, only the proportion of TAG content accumulates in TOR inhibited cell and not the total lipid content. Additionally, we could observe a negative correlation between protein concentration and starch/TAG amount suggesting that TOR inhibition affect carbon flux.

### Proteome and Phosphoproteome Analysis of Rapamycin-Treated *Chlamydomonas* Cells in Comparison With Control Samples

Proteome and phosphoproteome were analyzed by an LC-MS shotgun technique as previously described (Wienkoop et al., 2010; Valledor et al., 2013, 2014; Roustan et al., 2017). Proteins were extracted from five biological samples (three biological samples for phosphoproteomics) treated with 500 nM rapamycin or with the drug vehicle and harvested at 0, 24, and 48 h time points. Proteome datasets were defined based on proteins which were quantified in at least 4 biological replicates, in at least one class of samples (Supplementary Table S1). A total of 916 proteins were relatively quantified. To generate the phosphopeptide data matrix, the similar workflow was applied as in the recently published phosphoproteomic dataset obtained for nitrogen stress and recovery in *Chlamydomonas* (Roustan et al., 2017). Only peptides that had a phosphorylation localization probability > 0.75 and score difference > 5 were kept (Supplementary Table S2) resulting in 5283 identified phosphosites. Additionally, identified and quantified phosphopeptides were filtered according to the total protein dataset. After filtering, a data matrix of 1311 phosphopeptides mapped to 684 proteins was obtained (Supplementary Table S2). 1282, 25, 4 phosphopeptides that had single, double, triple phosphorylation sites, respectively, were detected. The distributions of phosphorylated Ser, Thr and Tyr residues were 1084, 185, and 42 respectively. Those distributions are comparable to previous studies (Wang et al., 2014; Roustan et al., 2017). According to (Valledor et al., 2014), the mercator online tool was used for protein and phosphoprotein annotation based on various organism-specific databases (Lohse et al., 2014).

### Proteome Differences Between Rapamycin and Control-Treated Samples

**FIGURE 2:**
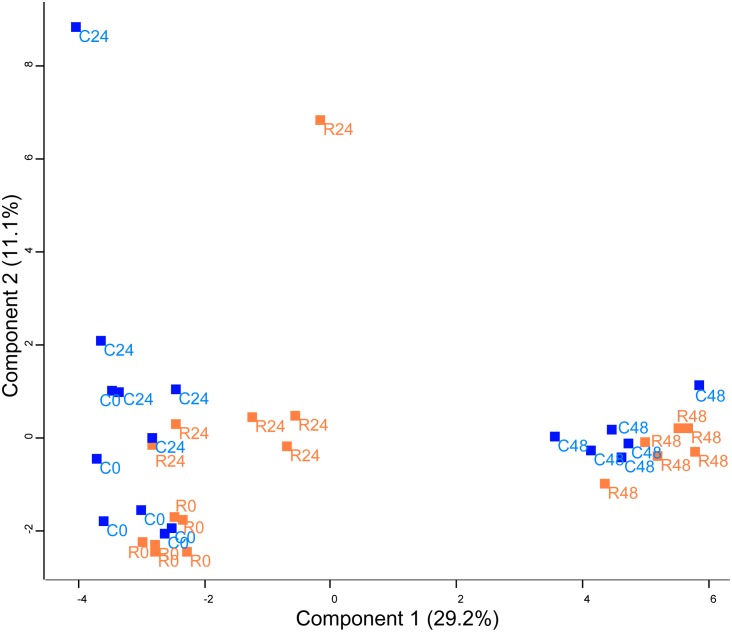
Principal component analysis (PCA) of protein abundance in *Chlamydomonas* cells treated with rapamycin or drug vehicle during a time course experiment (0, 24, and 48 h). The PCA includes all quantified proteins. Dots are the biological replicates (*n* = 5–6), control samples are colored in blue and rapamycin samples are colored in orange.

**FIGURE 3:**
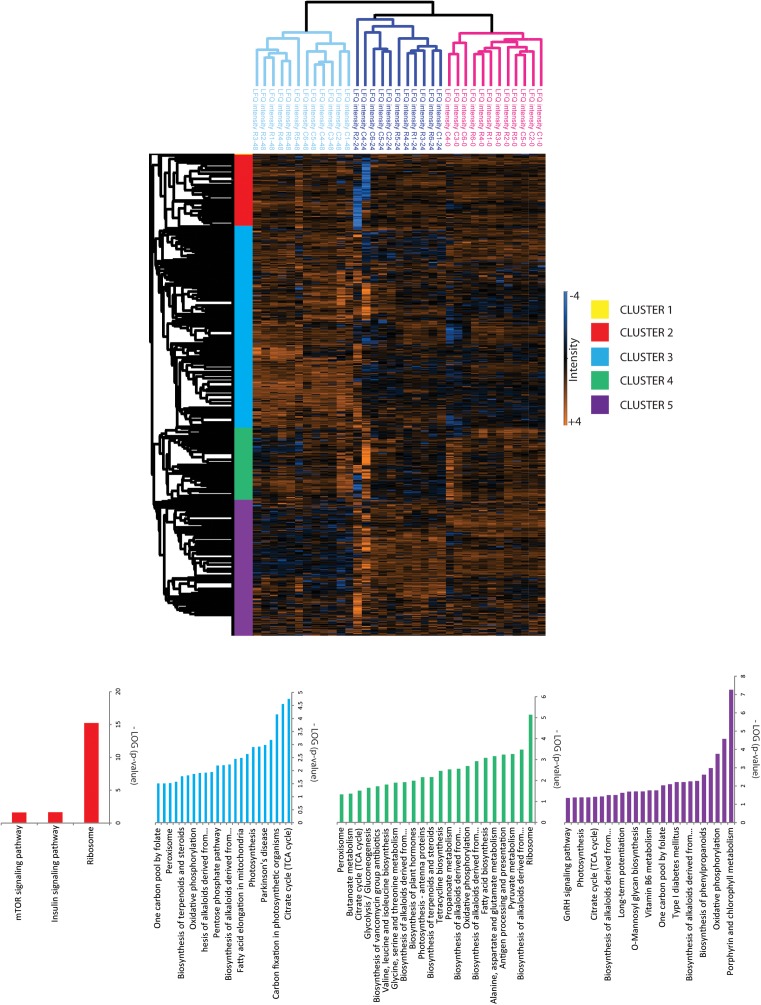
Hierarchical cluster analysis of quantified proteins. Clustering of proteins and samples was done based on Euclidian correlation and average linkage with Perseus. Clusters were exported in Supplementary Table S1 and KEGG enrichment analysis was done with the Algal functional annotation tool (Lopez et al., 2011) for each cluster as referred in Supplementary Table S1.

The heterotrimeric TOR complex is known to regulate translational activity and autophagy. To this end, TOR interacts with ribosomal S6 Kinase, and activates, by a phosphorylation cascade, RPS6 proteins and eukaryotic initiation factor 4B (Dobrenel et al., 2016a; Roustan et al., 2016; Perez-Perez et al., 2017). On the other hand, TOR complex phosphorylates ATG1 and inhibits autophagy. Orthologs for TOR, S6K, RPS6, and ATG1 proteins were previously found in *Chlamydomonas* (Roustan et al., 2016). A recent study in *Chlamydomonas* highlighted the nitrogen and concanamycin (translation inhibitor) effects on ATG8 and RPS6 protein content (Couso et al., 2017) whereas proteome adaptation to TOR inhibition remained unknown. To understand the global proteome acclimation of *Chlamydomonas* during rapamycin treatment, *in vivo* label-free shotgun proteomics was used to generate a proteome dataset. Protein changes between rapamycin treated samples and control samples were analyzed with principal component analysis (PCA) and hierarchical clustering (HCA) (Figures 2, 3). Identified protein clusters from HCA were used to perform a KEGG pathways enrichment analysis with the Algal Functional Annotation Tool (AFAT) (Lopez et al., 2011, 2014). Finally, the STRING database was used to understand the protein-protein interaction between the proteins identified as strictly affected by the two-way ANOVA treatment factor results (Table 1).

**Table 1:** Summary of the 61 proteins identified as strictly regulated by rapamycin treatment.

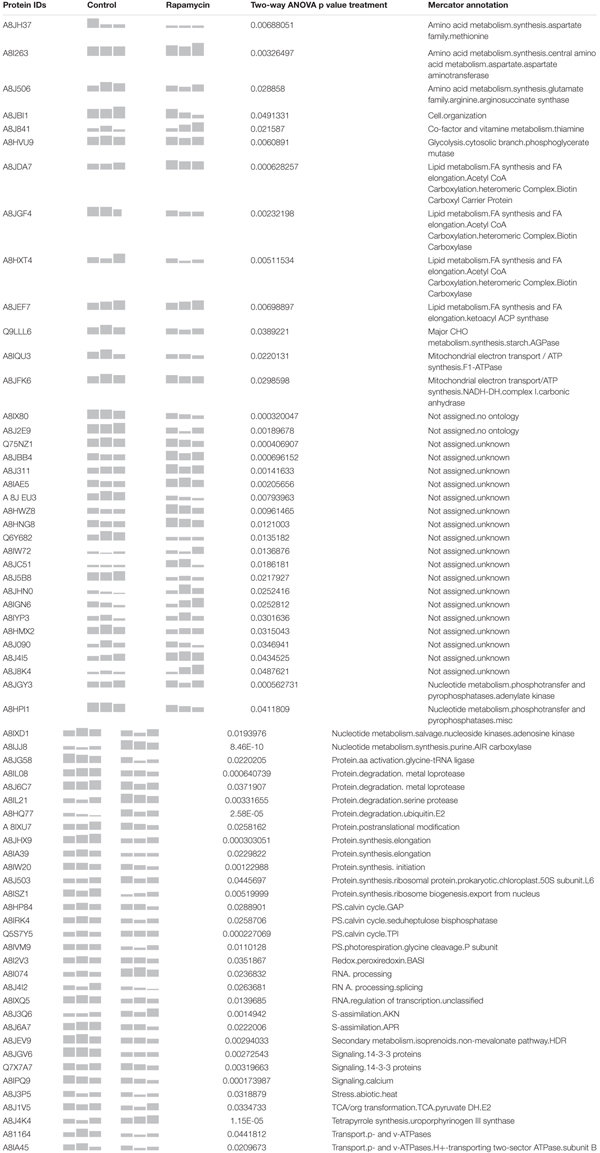

Unsupervised PCA (Figure 2) showed that principal component 1 (29.2% total explained variance) explained both the time course and rapamycin treatment highlighting also the strong effect of culture duration. Those results are in line with metabolomics findings (Juppner et al., 2017). The culture duration effect on the proteome is further strengthened by the HCA as indicated by the samples clustering (Figure 3). Protein cluster analysis indicates that growth inhibition induced by TOR treatment does not require a complete remodeling of the proteome but rather specific processes. While no significant enrichment was obtained for cluster 1, cluster 2 was enriched in proteins related to mTOR signaling pathway, insulin pathway, and ribosomes. Cluster 3 regroups proteins with a time-dependent up-regulation pattern. Enriched categories in cluster 3 were related to fatty acid elongation in mitochondria and peroxisome. Cluster 4 and 5 encompass proteins function predominantly related to photosynthesis, as well as primary and secondary metabolism (Figure 3).

**FIGURE 4:**
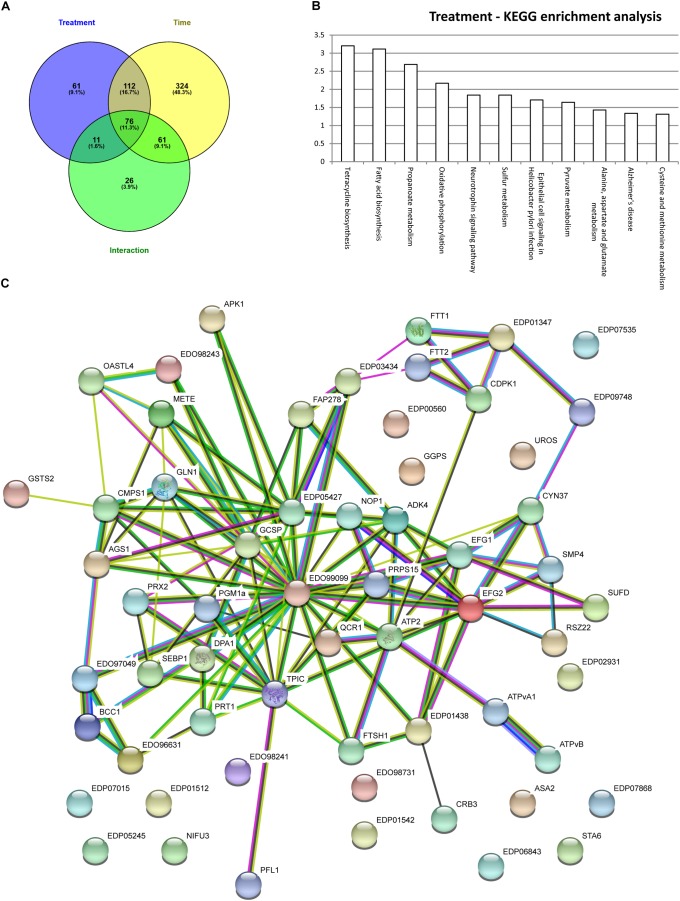
Graphic display of enriching fraction of KEGG pathways and protein–protein interaction network from proteins significantly regulated by the rapamycin. **(A)** Venn diagram with the two-way ANOVA results **(B)** KEGG enrichment analysis done with AFAT for the 61 proteins specifically regulated by rapamycin **(C)** STRING protein–protein interaction network performed with significantly changed 61 protein subset. Protein-Protein interaction network is based on evidences (threshold based on medium confidence score). For details, see Experimental Procedures, as well as Supplementary Table S1.

In order to assess the synergistic effect of multiple variables on *Chlamydomonas* proteome (time, treatment and time × treatment); two-way analysis of variance (ANOVA) was used. Two-way ANOVA tests the effect of two independent factors (hours of culture vs. treatment) on the proteome. Thus, two-way ANOVA distinguishes the influence of each independent factor on the proteome but also determines if they significantly interact. In total, 260 proteins were significantly affected by the rapamycine treatment, 587 proteins were significantly changed across the time course and 172 proteins were found to be significantly affected by the interaction of both factors (Figure 4A). Temporal effects on proteome, independent of the rapamycin treatment, were first investigated. To capture a trend in which quantified proteins showed a predominant effect of time dependency and a minimal effect of treatment; proteins with the highest *p*-value for both interaction and rapamycin treatment and the lowest *p*-value for time factor were selected. Most of the identified proteins through this screen were related to chloroplast metabolism (Supplementary Table S1), confirming the absence of significant changes observed in the photosynthetic apparatus and total chlorophyll content between the two treatments (Supplementary Figure S1). Furthermore, the two-way ANOVA established that some proteins were significantly changed by both, treatment and time factors (Figure 4A). Only 26 out of the 172 proteins were not overlapping with time or treatment factors. All the 26 proteins present inverse pattern between rapamycin and control treatment. Among them, a phosphoglucan water dikinase (A8J6C3) involved in starch degradation decreases up to 2-fold over the rapamycin treatment in contrast to the control cells. Further, abundance pattern of an isocitrate lyase (A8J244) involved in the gluconeogenesis declines in control sample, while it shows a 2.5-fold increase during the rapamycin treatment. Isocitrate lyase and phosphoglucan water dikinase protein changes corroborate with induced starch accumulation observed in rapamycin-treated cells (Figure 1E). While 260 proteins are significantly different between control and rapamycin-treated samples, only 61 proteins were specific to the treatment factor as summarized in Figure 4A. Interestingly, enrichment analysis of these 61 proteins points to sulfur, cysteine and methionine metabolic functions also recently shown to be involved in TOR signaling or affected by TOR inhibition in Arabidopsis (Figure 4B) (Dong et al., 2017). Similarly, proteins related to primary metabolism were enriched, such as amino-acid (A8I263 and A8J506), pyruvate (A8J1V5, A8HMX2, A8HXT4) and lipid metabolism (A8JDA7, A8JGF4, A8HXT4, A8JEF7) (Figure 4B).

Moreover, PPI-network with protein sequences of each of the 61 proteins discussed above was generated with STRING database (Figure 4C) (Franceschini et al., 2013). PPI-network analysis highlighted additional relevant proteins. In line with transcriptomics data (Ramundo et al., 2014; Kleessen et al., 2015), tetrapyrrole metabolism related protein such as uroporphyrinogen-III synthase involved in tetrapyrrole biosynthesis (A8J4K4) showed a 2-fold increase during rapamycin treatment compared to control cells. In contrast, proteins related to Calvin-cycle and photorespiration, such as A8HP84, A8IRK4, Q5S7Y5, and A8IVM9, were found to be down-regulated during rapamycin treatment. As well, several proteases were found to be differentially regulated during the rapamycin treatment. For instance, metalloprotease (A8IL08 and A8J6C7) predicted to be localized at the thylakoid membrane was down-regulated, while a serine protease (A8IL21) showed up to 2-fold accumulation during rapamycin treatment. Further, cell cycle-related protein CRB3 (Q6Y682) involved in circadian rhythm was 50% decreased compared to control samples. Interestingly, proteins related to the endomembrane systems such as the vacuole and the endoplasmatic reticulum (ER) were also affected by rapamycin treatment. For example, vacuolar ATP synthase subunits (A8I164 and A8IA45) were found to be significantly downregulated during rapamycin treatment compared to control cells. As well, 14-3-3 proteins (A8JGV6 andQ7X7A7) or CDPK2 homologs (A8IPQ9) have been shown to be located in the ER and present a significant downregulation during rapamycin treatment. As well, proteins related to mitochondria such as ATP synthase (A8IQU3), gamma carbonic anhydrase (A8JFK6) and a (D)-2-hydroxyglutarate dehydrogenase (A8J2E9) were down-regulated. Nevertheless, a mitochondrial ubiquinol-cytochrome c oxidoreductase subunit (A8JC51) increased over rapamycin treatment. Similarly, proteins related to purine metabolism were found to be significantly up-regulated during rapamycin treatment (A8JGY3, A8HPI1, A8IXD1, and A8IJJ8).

Finally, we also investigated the 199 other proteins presenting a significant difference in treatment factor, but also with time and interaction factors. Previously published transcriptomics data corroborate a significant decrease of proteins involved in pyrimidine biosynthesis (A8IMN5 and A8JIR0) (Ramundo et al., 2014). Moreover, mitochondrial chaperonin proteins were found to be up-regulated during rapamycin treatment (A8IMK1 and A8JES1).

### Phosphoproteome Profiling of Rapamycin vs. Control-Treated *Chlamydomonas* Samples

**FIGURE 5:**
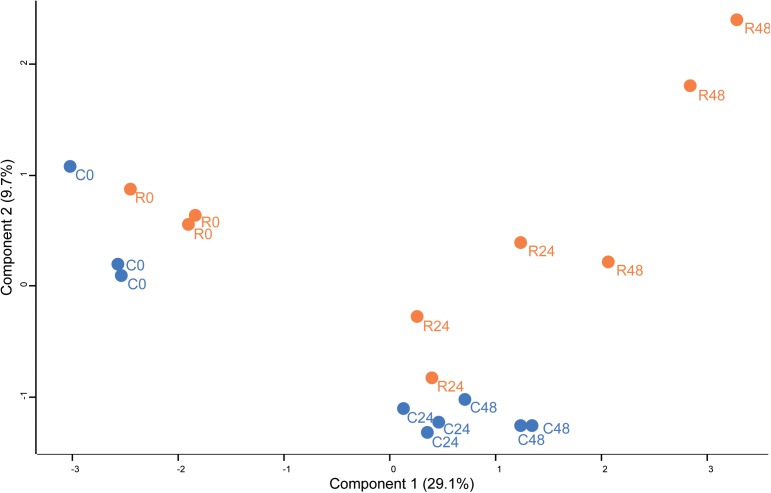
Principal component analysis (PCA) of phosphopeptides abundance in *Chlamydomonas* cells treated with rapamycin or drug vehicle during a time course experiment (0, 24, and 48 h). The PCA includes all phosphopeptides. Dots are the biological replicates (*n* = 3), rapamycin treated samples are colored in orange while control samples are colored in blue.

**FIGURE 6:**
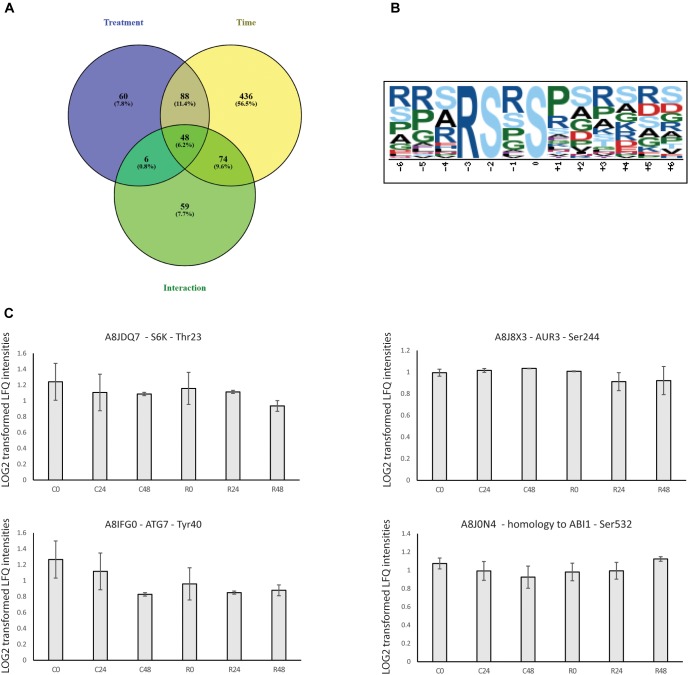
Graphic display of proteins significantly regulated by the rapamycin and overrepresented phosphorylation motif. **(A)** Venn diagram with the two-way ANOVA results **(B)** Motif enrichment analysis was done with Motif-x software with settings: >20 occurrences, significance threshold was set to 0.000001 and background proteome was *Chlamydomonas* UniProt database centered on Serine residues. **(C)** Relative abundance of TOR pathway-related phosphopeptides in control and rapamycin-treated cells sampled at 0, 24, and 48 h. Values represent means of three biological replicates and standard deviation.

Proteomics, metabolomics and transcriptomics data show the central role of TOR signaling in the regulation of *Chlamydomonas* growth. Analysis of proteomics data revealed that TOR inhibition elicits specific changes in a core set of proteins (Figure 4C). How TOR inhibition triggers those specifics change, is a remaining question. Since it was shown in mammals that rapamycin treatment inhibits TOR kinase activity (Jacinto et al., 2004), we take advantages of phosphoproteomics methods to decipher which signaling and regulatory mechanism could be controlled directly or indirectly by TOR kinase. Phosphopeptides changes were analyzed by PCA and two-way ANOVA using the dataset for control and rapamycin-treated samples. Phosphoproteome PCA analysis shows similar results as for the proteome. In both cases, we could differentiate treatment at each time point (Figure 5), however, both growth conditions presented similar trajectory, indicating the time-dependent effect on the data (all loading values can be found in Supplementary Table S2). In total two-way ANOVA identified 202 phosphopeptides significantly affected by treatment, 646 phosphopeptides significantly changed across the time course, and 187 phosphopeptides found to be significantly affected by the interaction of both factors (Figure 6A).

Table 2 present the 60 phosphopeptides specific to the treatment factor. Among them, A8J8X3 encodes an Aurora-kinase which is a member of a family of Ser/Thr kinases. Arabidopsis Aurora-kinase homolog is involved in the control of cell cycle, through its interaction with plant-specific cytoskeletal structures and its activities peak during cell division (Demidov et al., 2014). A8J8X3 phosphorylation site at Ser 244 is inhibited by about 15 % during the rapamycin treatment (Figure 6C). In line with cell cycle regulation, A8IZM6, and A8IFQ9 presented 2-fold lower phosphorylation level compared to control samples (Table 2). A8IZM6 encodes a homolog of human Lysine-Specific Demethylase1 involved in H3K4 methylation of flowering time loci (FLC and FWA) and A8IFQ9 a DNA binding protein with a nuclease activity which is involved in response to singlet oxygen in Arabidopsis (Jiang et al., 2007; Chen et al., 2015). Subsequently, RNA processing related proteins such as A8HYQ9, an RNA binding protein involved in photoperiodism and flowering in Arabidopsis, showed about 20% de-phosphorylation, at its phosphosite Thr 246, during TOR inhibition (Table 2) (Kim et al., 2016). Finally, several proteins related to cell signaling like a protein phosphatase 2C family protein (A8J7H2) and a calmodulin-like domain protein kinase (A8HSJ3) showed decreased phosphorylation level (Table 2).

**Table 2:** Summary of the 60 phosphosites identified as strictly regulated by rapamycin treatment.

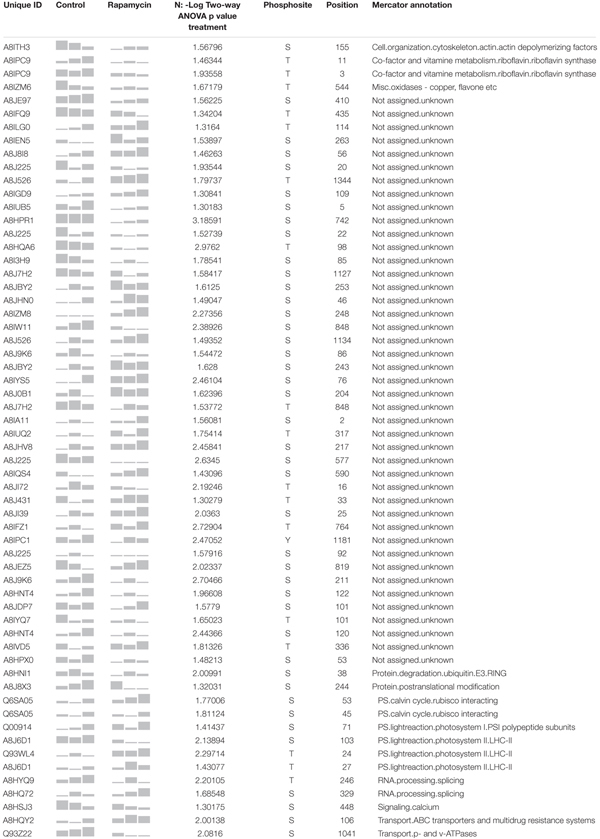

Further investigation was extended to TOR related proteins. For instance, A8JDQ7 encodes a protein-serine kinase, homolog to AtS6k that phosphorylates ribosomal protein *in vitro*. S6K is involved in translational up-regulation of ribosomal proteins (Mahfouz et al., 2006; Tzeng et al., 2009; Son et al., 2016). *Chlamydomonas* S6K homolog protein presents a phosphorylation at Thr 23 which significantly decreased down to 20% in rapamycin-treated cells compared to control samples (Figure 6C). As well, a homolog of ABI1 (A8J0N4) Protein phosphatase 2C-like which regulates the activation of the Snf1-related kinase OST1 via abscisic acid presented significantly increased phosphorylation level on Ser 532 (20% higher than control at 48 h) (Sridharamurthy et al., 2014). Lastly, an ortholog of human AUTOPHAGY 7 (A8IFG0), an activator of AUTOPHAGY 8, was found to be rapidly dephosphorylated at Tyr 40, with a phosphorylation level oscillating between 6 and 40% of the control samples. Interestingly, the Tyr 40 phosphosite is conserved between plants and mammals, however, its function is still unknown (Tanida et al., 2001).

Finally, to identify putative signaling pathways involved in TOR inhibition acclimation, we searched for over-represented sequence motifs in the 191 significantly changed phosphopeptides, from the treatment factor, by performing a motif-x analysis^1^. One motif was significantly enriched and present in 60 phosphopeptides, representing 42 proteins (Figure 6B). RSXS motif presents a 9.33-fold increase (Figure 6B) and has a serine at position 0, a basic arginine at position -3 followed by a serine at position -2. This motif is known to be targeted by 14-3-3 proteins (Johnson et al., 2010). At the proteome, both 14-3-3 proteins were found to be inhibited by rapamycin treatment (Table 1).

## Discussion

**FIGURE 7:**
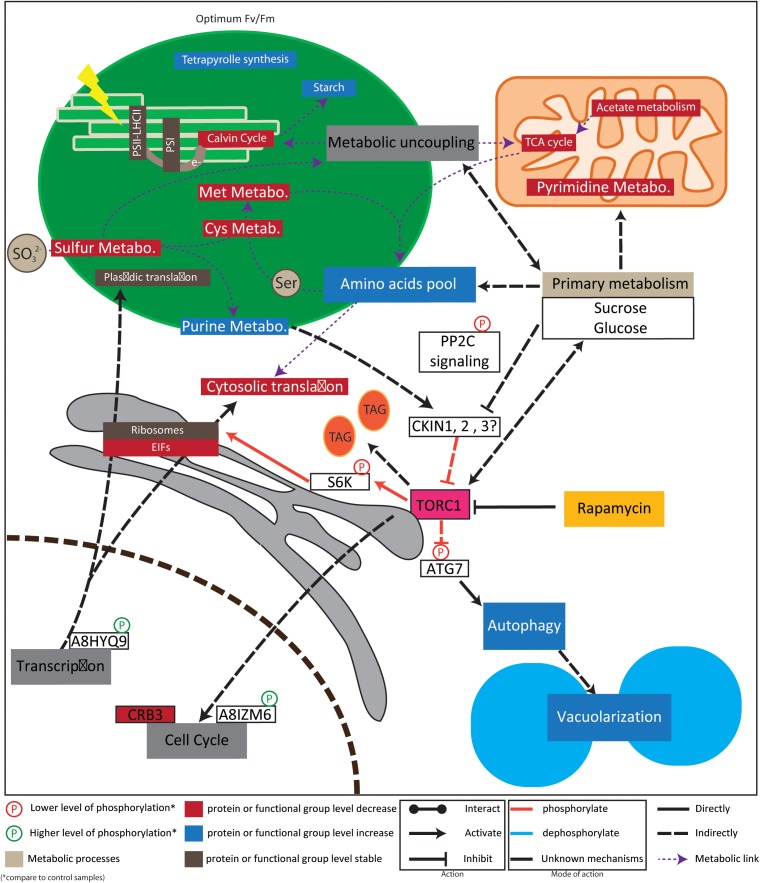
Model for regulation of rapamycin treatment response. Intrestingly, our data suggest that chloroplastic function are not inhibited but rather adapted. Especially, plastid localized, Calvin cycle, sulfur, cystein and methionine related proteins are down regulated. Instead, starch accumulate and purine metabolsime is stimulated. Those observation are line with previous reports indicating a connection of sulfur metabolism with TOR signaling (Dong et al., 2017). Together with our results, recent metabolic data suggest also a metabolic uncoupling between the plastid and mitochondria, associate with a decrease of mitochondrial metabolic activity (Juppner et al., 2017). In line with plastid maintenance, ribosomes didn’t present clear regulation pattern, instead EIF factors were down regulated. Reduced cytosolic translation activity is in line with S6K phosphorylation previously shown to be responsible for translation regulation (Dobrenel et al., 2016b). This reduced cytosolic translation activity could explain amino acid accumulation previously detected (Juppner et al., 2017). Further, protein associated to circadian cycle and flowering time regulation were together decreasing in protein abundance or phosphorylation level. Finally, phosphorylation of ATG7 is in line with previously detected autophagy induction and cell vacuolarization (Crespo et al., 2005; Couso et al., 2017). For example, the red- arrow between TOR and SK6 indicate that TOR complex directly and positively regulates RPS6 by phosphorylation. PS is photosystem; LHCII is Light-Harvesting complex-II; e- represents electron from the electron transfer chain; TAG stands for tri-acy-glycerol; Cys for cysteine; Met for methionine; for protein accession number and name refer to results part.

In human, yeast, and Arabidopsis, TOR kinase forms a heterotrimeric complex with LST8 and RAPTOR which plays a central role in energy sensing and signaling. Indeed, upon sufficient level of energy and nutrients the TOR pathway stimulates cell growth through translation and transcription stimulation while it is inhibiting autophagy process (Roustan et al., 2016). In Arabidopsis, data strongly support a specific acclimation of TOR and its counterpart AMPK signaling pathway (Nukarinen et al., 2016). It was previously shown that *Chlamydomonas* cells treated with rapamycin present growth inhibition, which was abolished in a *fkbp12* mutant line (Crespo et al., 2005). As well, several phylogenetic studies have identified orthologs of TOR and AMPK pathways, in *Chlamydomonas* (Roustan et al., 2016). However, a similar loss of genes, as for Arabidopsis, point toward a specific regulation of the energy sensing and signaling in plant kingdom (Roustan et al., 2016). Unlike Arabidopsis, fewer investigations have been conducted in *Chlamydomonas* to unravel TOR function and none of them have performed an *in vivo* label-free phosphoproteomics analysis. In this context, our work aimed to compare the proteome and the phosphoproteome of *Chlamydomonas* cells treated with rapamycin or drug vehicle (control) at 0, 24, and 48 h in continuous light to identify metabolic processes at the protein level involved in TOR inhibition acclimation. To gain further understanding, physiological parameter such as growth rate, photosynthesis, protein content and carbon storage (starch and lipids) were measured along the proteome and phosphoproteome. From all detected proteins we quantify 916 protein and 1312 phosphopeptides across all time point. We used multivariate statistics to investigate the dynamic acclimation of both proteome and phosphoproteome to rapamycin treatment. Both a strong time effect as well as a significant effect of rapamycin treatment were observed in the data (Figure PCA and two-way ANOVA). These results are summarized in a proposed model of Chlamydomonas acclimation to rapamycin treatment as shown in Figure 7.

### Rapamycin Treatment Concomitantly Inhibits Cell Growth and Induced Changes in Cell Cycle Related Proteins

Here, we measured the phenotypic acclimation of *Chlamydomonas* in response to 500 nM of rapamycin. We found that this rapamycin concentration was sufficient to inhibit cell growth, but to a lesser extent that it was previously reported (Crespo et al., 2005). According to this observation, recent studies on *Chlamydomonas* acclimation to rapamycin, have used a higher concentration of rapamycin (Juppner et al., 2017). Consequently, most of the significant changes occurring at the physiological level were observed at 48 h after the treatment, even if a clear trend could be observed in earlier time points. As already reported we did not found a total inhibition of the cell growth during rapamycin treatment at both cell number and fresh weight level. Previously, cell-cycle of *Chlamydomonas* cells treated with rapamycin was found to present a delay compared to the control (Juppner et al., 2017). Those results indicated that TOR could also be involved in cell cycle regulation. Coincidently, CRB3, involved in the coordination of the cell cycle with the circadian rhythm was downregulated in our data (Niwa et al., 2013). Similarly, other proteins involved in regulation of flowering time loci or in cytoskeletons organization presented de-phosphorylation (Figure 7). Altogether our data suggest that TOR kinase coordinate the progression of the cell cycle with nutrient availability and protein translation (Dong et al., 2017; Werth et al., 2018).

### Rapamycin Treatment Significantly Changes Phosphoprotein and Protein Dynamics of Evolutionary Conserved TOR – Targets

Among the proteins and phosphosites measured in our approach, we could find proteins related to TOR signaling. For instance, the evolutionary conserved S6K protein was identified in our phosphoproteomics screen. Together with TOR, S6K is known to be involved in the regulation of protein translation. In line with this hypothesis we found a significant decline of the Thr 23 phosphosite of the S6K which is a TOR target (Figure 7). There is, however, no conservation of this particular phosphosite found between mammals, yeast and plants.

Other proteins potentially involved in TOR downstream translational control and targets of S6K are RPS6 proteins (Nukarinen et al., 2016). While we did not detect phosphorylation levels of RPS6 protein, our proteomic data showed that RPS6 protein level are stable over the treatment. These results are in line with a recent investigation of the early response of the *Chlamydomonas* phosphoproteome to rapamycin treatment (Werth et al., 2018). More precisely, this study investigated the RPS6 phosphorylation at the conserved Ser242 site but could not detect any change in the phosphorylation level (Werth et al., 2018). This suggests that S6K protein is targeting other RPS6 phosphorylation sites or other ribosomal proteins in *Chlamydomonas* or functions via different mechanisms At the proteome level, rapamycin treatment seems to affect eukaryotic initiation factors rather than cytosolic ribosome proportion (Supplementary Table S1). Similarly, eukaryotic initiation factors were found to be affected by nitrogen availability (Valledor et al., 2014; Roustan et al., 2017).

### Analysis of Proteins Related to Carbohydrate and Lipid Storage Pool Metabolism Suggest That Starch and TAG Accumulation Is Related to Carbon Flux Re-rooting Rather Than *de novo* Biosynthesis

According to our and others observations rapamycin treatment in *Chlamydomonas* affects protein translation via TOR inhibition. Since translation accounts for more than 60% of energy consumption, it is necessary to re-route energy toward storage compound synthesis (see Figure 1). In our study, decreasing protein content coincides with the increase of starch by approximatively 50%, while the lipid content stays stable over the time. However it appears that the lipid composition is changing. Investigation of the lipid composition by thin-layer-chromatography revealed a higher TAG amount during rapamycin treatment (Supplementary Figure S1). Lipid, starch and protein content are therefore in line with previous publications (Imamura et al., 2015, 2016; Couso et al., 2016; Mukaida et al., 2016; Juppner et al., 2017). How TAG accumulates during rapamycin treatment is of importance since TAG molecules are of a major interest for general lipid and biodiesel production (Merchant et al., 2012; Liu and Benning, 2013; Furuhashi et al., 2016). At the proteome level we observed a complex dynamic pattern. Among the significantly affected proteins, three are part of the acetyl coenzyme A carboxylase complex involved in the lipid biosynthesis. Those proteins are A8JDA7, a Biotin Carboxyl Carrier Protein (BCC1), A8JGF4, a Biotin Carboxylase and A8HXT4 a Pyruvate carboxylase (PYC1). As well two other chloroplastic proteins, A8JEF7 3-ketoacyl-CoA-synthase (KAS1) and A8IQB8, a Plastid acyl-ACP desaturase (FAB2), involved in lipid elongation and desaturation. Whereas the proteins of fatty acid synthesis showed a slight but significantly decreased level FAB2 had increased levels (Supplementary Table S1). FAB2 has been shown to be involved in the alteration of the chloroplast membrane in response to copper deficiency increasing the level of desaturation (Castruita et al., 2011). Altogether the data indicate that TAG accumulation is rather linked to a carbon pool reallocation than to *de novo* synthesis. While recent studies have highlighted the central role of ER in TAG synthesis (Li-Beisson et al., 2013; Couso et al., 2017; Kim et al., 2018), those identified proteins in our study are predicted to be localized in the chloroplast or in the cytosol (Supplementary Table S1).

Also metabolic enzymes related to starch metabolism showed a complex pattern. A8J6C3, a Phosphoglucan water dikinase (PWD1) involved in starch degradation showed decreased levels in rapamycin treated cells compared to control samples. As for lipids starch might be also a storage compound balancing the over flow of reduced carbon under stress conditions.

While proteins of the photosystems PSII and PSI are stable over treatment (Supplementary Table S1), we also observed that Calvin cycle-related proteins were found to be downregulated during the rapamycin treatment (Figure 7). Such observation could indicate that chloroplast to nucleus retrograde signaling is adjusting the import of plastid related proteins in order to reduce the energy impute and avoid a ROS burst. In line with this hypothesis, a Protein disulfide isomerase (A8JBH7) and a Glutathione peroxidase (O22448) showed increased levels under rapamycin treatment compared to control samples. Altogether, those results correlate with the recent observation made in *tor* Arabidopsis mutant which showed a strongly affected photosynthesis (Dong et al., 2015).

### Cellular and Organellar Acclimation in Response to TOR Inhibition

Transcriptomic data investigating the TOR role for transcriptome regulation and its coordination with the metabolome were previously published (Kleessen et al., 2015). Those studies have indicated that TOR inhibition up-regulated genes involved in tetrapyrrole synthesis, vacuolar function, amino acids metabolism and transport as well as folding and chaperonin related genes. On the other hand, genes involved in nucleotide metabolism, cell cycle and DNA replication and repair, were down-regulated (Ramundo et al., 2014; Kleessen et al., 2015). In line with those studies, we have found similar changes in the proportion of proteins related to those functional groups. For instance, we have found increased amounts of tetrapyrrole related proteins, being in line with a slight increase of chlorophyll a/b ratio content during rapamycin treatment (Supplementary Figure S1B). It would be interesting to investigate the hypothesis that in absence of translation a part of the nitrogen pool is re-routed partially toward chlorophyll biosynthesis. Further, most of Calvin cycle-related proteins were found to be downregulated during the rapamycin treatment (Figure 7) indicating a potential adjustment of retrograde signaling in order to reduce the energy impute but also maintain nitrogen availability. Accordingly, we have observed a clear distinction between chloroplast acclimation during nitrogen depletion in our recent study (Roustan et al., 2017) and rapamycin treatment (this study), especially with the focus on chlorophyll content. Under nitrogen starvation there is a significant drop of chlorophyll in the first 48 h in contrast to the rapamycin treatment (Figure 1 in Roustan et al., 2017, and Supplementary Figure S1 this study). This indicates very different acclimation strategies of the chloroplast organelle to TOR inhibition either induced by rapamycin or nitrogen depletion. Future studies will address this question in more detail.

### Rapamycin Treatment Induces Changes in Sulfur and Nucleotide Metabolism

Several proteins related to amino acid metabolism were found as differentially regulated during the rapamycin treatment. Among them, proteins related to serine, cysteine and methionine metabolism as well as to sulfur metabolism attracted our attention. Indeed, those three amino acid biosynthetic pathways are located in the chloroplast and are interconnected with sulfur metabolism. Sulfur metabolism itself was recently related to TOR signaling (Dong et al., 2017). At the sulfur metabolic level, an adenosine-5-phosphosulfate-kinase (A8J3Q6 - AKN) was found to be 2-fold upregulated during the rapamycin treatment while an adenosine 5-phosphosulfate reductase (A8J6A7) was found to be downregulated. Further, A8IH03, a phosphoserine aminotransferase which is involved in serine biosynthesis in the chloroplast and operates via the phosphorylated pathway presents higher abundance in the rapamycin-treated cell than in the control. Serine is then metabolized into *O*-acetyl serine (OAS) which is a limiting precursor for sulfur assimilation. The enzyme involved in the interconversion of OAS to cysteine, the *O*-acetylserine (Thiol)-lyase (A8ISB0 - OAS-TL B) was stable during rapamycin treatment while it increased in control cells. Concomitantly, we observed that proteins related to methionine biosynthesis such as an *S*-adenosyl-l-homocysteine hydrolase 2 (SAHH2) (A8IXE0), a plastidic methionine synthase (A8JH37) as well as a methionine adenosyltransferase (A8HYU5) were down-regulated during the rapamycin treatment (Figure 7). Recently, a metabolic study has observed that increased levels of cysteine displayed significantly lower fold upshifts compared to serine (Juppner et al., 2017). Additionally, in the same study authors have found that purine related metabolites are more upregulated than pyrimidine related ones (Juppner et al., 2017). Both, purine and sulfur metabolism are interconnected since the AKN protein is also involved in the purine metabolism (Herrmann et al., 2015). Concerning the nucleotide metabolism, we observed an increase of enzymes involved in the synthesis of *de novo* purine biosynthesis such as A8J3Y6 (PUR4 a formylglycinamidine ribonucleotide synthase), A8I6R4 (PUR5 a phosphoribosylformylglycinamidine cyclo-ligase), A8JBQ5 (PUR7 a SAICAR synthetase) A8IJJ8 (AIR carboxylase) A8IVF0 (AICAR transformylase). Additionally, we could confirm the downregulation of pyrimidine metabolism represented by two carbamoyl phosphate synthetases (A8IMN5, A8JIR0) and a translation factor (A8HTK7) (Figure 7).

Altogether, data suggest a strong interplay between TOR signaling, sulfur metabolism and nucleotide metabolism, especially de novo purine biosynthesis. Interestingly, all those signaling pathways are located in the chloroplast. Remarkably, it was previously observed in *Chlamydomonas* that cysteine and methionine metabolites were strongly affected in line with transcriptomics data (Kleessen et al., 2015). Our results were concordant also with the observed downregulation of sulfur assimilation enzymes. In Arabidopsis *tor* mutants, sulfur-related metabolism was shown to be impaired (Ren et al., 2012; Caldana et al., 2013). More recently, it was shown in Arabidopsis, that sulfur availability regulates plant growth via the glucose-TOR signaling pathway (Xiong et al., 2013; Dong et al., 2017). Further, it was previously observed in animals and yeast that cell lines treated with AICAR metabolite presented enhanced AMPK activity (Corton et al., 1995; Sakamoto et al., 2004). It would be of interest, to test whether AICAR could also stimulate *Chlamydomonas* AMPK pathway.

Finally, data suggest that mitochondrial metabolism is responsive to TOR inhibition. Indeed, we observed an inhibition of carbonic anhydrases (A8IT01, A8J4Z8, and A8JFK6), TCA cycle-related enzymes (A8HMQ1, A8JHU0, A8J1V5, and A8IWJ8), and proteins involved in mitochondria electron transfer and ATP synthase (A8IVJ7 and A8IQU3). It is known that TCA cycle is at the crossroad between amino acid metabolism and energy production in mitochondria and is fueled in part by sugar and amino acid metabolism. For instance, carbonic anhydrases are low-CO_2_ responding protein and their downregulation is in line with changes observed at the chloroplast level. Together this suggests that sudden TOR inhibition triggers an over-accumulation of carbon and energy, which is reflected by downregulation of mitochondrial metabolism and TCA cycle (Figure 7) (Ramanathan and Schreiber, 2009).

### Is TOR Involved in Endomembrane System Regulation?

Interestingly, several proteins involved in endomembrane system trafficking or function were found. Regarding rapamycin treatment, several studies have linked translation and lipid metabolism with ER, vacuole, and autophagy (Perez-Perez et al., 2017). In *Chlamydomonas*, a vacuolarization process was observed during rapamycin treatment, in line with an increase of autophagy process and ATG8 accumulation (Crespo et al., 2005; Couso et al., 2017). In our dataset, vacuolar ATPase subunits were decreasing over the rapamycin treatment. Activation of autophagy is driven by dephosphorylation of the phosphosite Y40 of AUTOPHAGY 7, an activator of AUTOPHAGY 8, by about 40% compared to control samples. Mechanistically, it is known that TOR is inhibiting autophagy via protein phosphorylation of ATG1 in human (Kim et al., 2011). Additionally, the vacuole is known to be a central player of protein turn-over (Kim et al., 2001). In a recently published metabolomics dataset, the rapid accumulation of most amino acids were found to occur in the first 30 min (Juppner et al., 2017). In this context, we observed that most of the enzymes related to amino-acid metabolism were down-regulated. Altogether these data point toward a role of TOR in the protein turn-over regulation. Lastly, several proteins localized and/or related to endoplasmatic reticulum metabolism were also downregulated during a rapamycin treatment. For example, 14-3-3 proteins and the CDPK 2 protein were already found to be located to the ER (Voigt et al., 2004). Further, PDIL protein was in contrast up-regulated as other Redox related enzymes (Supplementary Table S1), suggesting a redox unbalance.

In line with previously unraveled endomembrane dynamics under rapamycin treatment, we found here several proteins involved in the Endosomal Sorting Complex Required for Transport (ESCRT) machinery (VPS4, VPS60, and VPS46) to be significantly upregulated by the rapamycin treatment. It would be of a major interest to further investigate the endomembrane system as well as the Endosomal Sorting Complex Required for Transport (ESCRT) in *Chlamydomonas*. Indeed, biochemical fractionation of *Chlamydomonas* cells suggests that TOR and LST8 are co-localized with membranous sites (Díaz-Troya et al., 2008a).

## Conclusion

Energy is at the heart of life and is the most important evolutionary driver. In order to continuously adjust the cell activity to the available energy, the TOR and AMPK pathways are regulating catabolic and anabolic processes. How plants TOR and AMPK pathways have specifically evolved to integrate plastid endosymbiosis is a remaining question (Roustan et al., 2016). Both TOR and AMPK are protein kinases. Therefore our main interest has been to investigate the dynamics of *in vivo* protein phosphorylation in response to TOR control. To address this question we have analyzed rapamycin treated *Chlamydomonas reinhardtii* cells with *in vivo* label-free shotgun phosphoproteomics in order to understand how single-cell green algae acclimate to TOR inhibition under continuous light. Our analysis points toward a central role of TOR in the control of the transcription and translation and therefore directly affecting the carbon flux and energy flux (Figure 7). Indeed, the analysis of transcriptomic, proteomic and metabolic datasets indicate a negative correlation between the accumulation of amino acids and the down-regulation of the associated biosynthetic enzymes. As well, differential regulation between pyrimidine and purine metabolism were found suggesting that TOR inhibition is affecting the nucleotide pool as found previously (Figure 7) (Juppner et al., 2017). In turn, it seems that the subcellular chloroplast-mitochondria synergy is uncoupled to avoid an overproduction of energy compared to a slow down of translational and growth processes (Figure 7). In line with such organelle acclimation, we found an accumulation of proteins related to the endomembrane trafficking system such as ESCRT-III related proteins. These data and the comparison with nitrogen starvation and recovery studies on the phosphoproteome, proteome and metabolome (Valledor et al., 2014; Roustan et al., 2017) force new research questions especially about organellar acclimation und subcellular compartmentation of metabolic changes, control of chloroplast-mitochondrium interaction and chloroplast nucleus retrograde signaling processes. In future we will address these questions with non-aqueous subcellular fractionation combined with proteomics and metabolomics (incl. lipidomics, Furuhashi et al., 2016) as well as metabolic network modeling (Nägele and Weckwerth, 2013, 2014; Nägele et al., 2014, 2016; Fürtauer et al., 2016; Nukarinen et al., 2016).

## Materials and Methods

### Plants Material and Growth Conditions

*Chlamydomonas reinhardtii* CC-503 *cw92*, mt+, agg1+, *nit1*, *nit2* cultures were grown in HEPES-Acetate-Phosphate medium supplemented with 7 mM NH_4_Cl (HAP; TAP medium in which Tris was replaced by 5 mM HEPES) at 25°C with shaking (120 rpm) in a 16:8 light:dark photoperiod (85 μmol m^-2^ s^-1^; Sylvania GroLux lamps). To start the experiments, cultures were pelleted down by centrifugation at the end of the night, washed two times with fresh HAP medium and re-suspended in HAP media treated with 500 nM rapamycin or with the drug vehicle to a final density of 1–3 × 10^6^ cells mL^-1^. From this point, cells were cultivated under continuous light. Cells were sampled at 0, 12, 24, and 48 h time points after transfer in the fresh medium supplemented or not with rapamycin for physiological parameter measurement. For proteomics and phosphoproteomics, cells were harvested at 0, 24, and 48 h.

### Physiological Measurements

Growth parameter (cell number and fresh weight), starch, lipid and photosynthetic parameters (Fv/Fm, total chlorophyll content and chlorophyll a/b ratio) were measured as previously described (Valledor et al., 2014; Roustan et al., 2017). Total protein content was determined with Bradford assay using BSA as a protein standard. Tri-acyl-glycerol content was monitored by thin-layer-chromatography as described. Shortly 5 mg fresh weight were load and separated on silica gel 60 (EMD Chemicals) using hexane-diethyl ether-acetic acid (90:7.5:1 [v/v/v]) and visualized under UV light after spraying with primuline (Sigma). (1 mg in 100 ml of acetone/water, 80/20, v/v) (Li et al., 2008).

### Protein Extraction and Phosphopeptide Enrichments

Total proteins from *Chlamydomonas* cell pellets were extracted by a phenol-phase extraction protocol as previously described (Roustan et al., 2017). Protein precipitation was performed by mixing the phenol fraction with 2.5 volumes of 0.1M ammonium acetate in methanol. After a 16 h incubation period at -20°C, the samples were centrifuged for 5 min at 5000 × *g*. The protein pellets were washed twice with 0.1M ammonium acetate, one time with acetone and air dried at room temperature. The protein pellets were dissolved in 8M urea/100 mM ammonium bicarbonate (AmBic) supplemented with protease and phosphatase inhibitor cocktails as indicated by the supplier (Roche, Cat. No. 05 892 791 001 and Cat. No. 04 906 837 001). Protein concentration was determined using the Bio-Rad Bradford Assay with BSA as a standard. 200 μg (proteomic)/500 μg (phosphoproteomic) of total protein per sample was first reduced with dithiothreitol (DTT) at a concentration of 5 mM at 37°C for 45 min. Cysteine residues were alkylated with 10 mM iodoacetamide (IAA) in darkness at room temperature (RT) for 60 min. Alkylation was stopped by increasing DTT concentration to 10 mM and incubating the samples in the dark at RT for 15 min. Then the urea concentration was diluted to 2 M with 50 mM AmBic/10 % acetonitrile (ACN). CaCl2 was added to a final concentration of 2 mM. Trypsin digestion (Poroszyme immobilized trypsin; 5:100 v:w) was performed at 37°C overnight. Protein digests were desalted with C18 solid phase extraction (SPE) (Agilent Technologies, Santa Clara, CA, United States) and carbon graphite SPE as described by Furuhashi et al. (2014) and both fractions were subsequently pooled and dried before LC-MS measurement in the case of proteomics or before phosphopeptides enrichment for phosphoproteomics approach. 5 mg of TiO2 (Glygen Corp.) was used to enrich phosphopeptide as described previously (Bodenmiller et al., 2007; Chen et al., 2010) and dried in a vacuum concentrator.

### LC-MS for Proteomics and Phosphoproteomics

#### For Proteomics

Samples were dissolved in 2% ACN and 0.1% FA to a final concentration of 0.2 μg equivalent total protein per μL. 1 μg equivalent total protein was loaded into a one-dimensional (1D) nanoflow LC-MS/MS system equipped with a precolumn (Eksigent, Germany). Peptides were eluted using an Ascentis column (Ascentis Express, peptide ES-C18 HPLC column (SUPELCO Analytical, Bellefonte, PA, United States), dimension 15 cm × 100 μm, pore size 2.7 μm) during a 120 min gradient from 5% to 50% (v/v) acetonitrile, 0.1% (v/v) formic acid. MS analysis was performed on an Orbitrap LTQ XL mass spectrometer (Thermo, Germany) with a controlled flow rate of 500 nL per minute. Specific tune settings for the MS were as follows: spray voltage was set to 1.8 kV; temperature of the heated transfer capillary was set to 180°C, full scan range 350–1800 m/z, FTMS resolution 120000. Each full MS scan was followed by ten MS/MS scans, in which the ten most abundant peptide molecular ions were dynamically selected, with a dynamic exclusion window set to 60 s. Ions with a + 1 or unidentified charge state in the full MS were omitted from MS/MS analysis. For injection control automatic gain control (AGC) for full scan acquisition in the Orbitrap was set to 5 × 10^5^ ion population, the maximum injection time (max IT) was set to 500 ms. Orbitrap online calibration using internal lock mass calibration on m/z 371.10123 from polydimethylcyclosiloxane was used. Multistage activation was enabled with neural losses of 24.49, 32.66, 48.999, 97.97, 195.94, and 293.91 Da for the 10 most intense precursor ions. Prediction of ion injection time was enabled and the trap was set to gather 3 × 10^4^ ions for up to 50 ms.

#### For Phosphoproteomics

Samples were dissolved in 11 μL of 2% ACN and 0.1% FA and 5 μL of the mixture was separated on an EASY-Spray PepMap RSLC 75 μm × 50 cm column (Thermo Fisher Scientific Inc., Waltham, MA, United States). Peptides were eluted using a 240 min linear gradient from 2 to 40% of mobile phase B (mobile phase A: 0.1% [v/v] formic acid (FA) in water; mobile phase B: 0.1% [v/v] FA in 90% [v/v] ACN) with 300 nL/min flow rate generated with an UltiMate 3000 RSLCnano system. Peptides were measured with an LTQ-Orbitrap Elite (Thermo) using the following mass analyzer settings: ion transfer capillary temperature 275°C, full scan range 350–1800 m/z, FTMS resolution 120000. Each FTMS full scan was followed by up to ten data dependent (DDA) CID tandem mass spectra (MS/MS spectra) in the linear triple quadrupole (LTQ) mass analyzer. Dynamic exclusion was enabled using list size 500 m/z values with exclusion width ± 10 ppm for 60 s. Charge state screening was enabled and unassigned and +1 charged ions were excluded from MS/MS acquisitions. For injection control automatic gain control (AGC) for full scan acquisition in the Orbitrap was set to 5 × 10^5^ ion population, the maximum injection time (max IT) was set to 200 ms. Orbitrap online calibration using internal lock mass calibration on m/z 371.10123 from polydimethylcyclosiloxane was used. Multistage activation was enabled with neural losses of 24.49, 32.66, 48.999, 97.97, 195.94, and 293.91 Da for the 10 most intense precursor ions. Prediction of ion injection time was enabled and the trap was set to gather 5 × 10^3^ ions for up to 50 ms.

### Data Analysis and Statistics

MaxQuant 1.5^2^ and the Andromeda search algorithm were used against the *Chlamydomonas* UniProt and JGI_236 version databases to perform peptide identification, phosphorylation site mapping and protein and phosphopeptide quantification (Cox and Mann, 2008; Cox et al., 2011). For both proteomics and phosphoproteomics data processing the following parameters were applied: Two and three missed cleavages were allowed respectively for the proteomics and phosphoproteomics analysis. Methionine oxidation and protein N-terminal acetylation were endorsed as dynamic modifications. For phosphoproteomics analysis, additionally, phosphorylation of serine, threonine and tyrosine residues was permitted to occur as dynamic modifications. Mass tolerance was set to 5 p.p.m. for parental ions and 0.8 Da for the MS/MS fragment. For both peptide and protein levels, false discovery rate was set to 1%. In both analysis peptide quantification was performed by peak integration at the MS1 level using Max Quant 1.5. For proteomics analysis the Label-free quantification of proteins was done with a peptide ratio count 2 according to the Max Quant recommendations. The mass spectrometry (Phospho)proteomics data have been deposited to the ProteomeXchange Consortium (Deutsch et al., 2017) via the PRIDE (Vizcaíno et al., 2016) partner repository with the dataset identifier PXD011489. Perseus 1.5 software, was used for further filtering and data processing (Tyanova et al., 2016). Proteomics data set was obtained by filtering the data matrix so that proteins that were identified in at least four biological replicates in at least one class of samples were included. Before PCA, values were log2 transformed and missing values were replaced by random numbers drawn from the normal distribution that represents low-abundance measurements of each sample. Phosphopeptide data were filtered as the total proteomics data on the base of three biological samples per sample class. Additionally, only phosphopeptides that passed the class I criteria (phosphosite probability > 75% and score difference > 5) were included in the final dataset (Olsen et al., 2006). Moreover, phosphopeptide abundance was normalized to the median of each sample, log2 transformed and missing values replaced using the same method as in total proteomics data. Further, PCA, HCA based on Spearman rank correlation and an average linkage of z-transformed phosphopeptide abundances, Analysis of variance (two-way ANOVA) was performed with Perseus software (Tyanova et al., 2016). Algal Functional Annotation tool (AFAT) was used to identify enriched functions from the protein cluster resulting from the HCA analysis or from the significantly changed proteins (Lopez et al., 2011). The motif-x analysis was used to identify phosphorylation motifs that were over-represented in our dataset^3^ (Schwartz and Gygi, 2005). The analysis was conducted on peptides which significantly changed in treatment factor from the two-way ANOVA analysis. Two searches were performed with a serine residue or a threonine residue as central position. Background proteome was *Chlamydomonas* UniProt database centered on S, T and Y amino acids. Protein-protein interaction networks were created using the STRING database for Known and Predicted Protein-Protein Interactions with the standard setting^4^ (von Mering et al., 2005).

## Author Contributions

VR and WW conceived the study. VR performed measurements, analyzed the data, and wrote and revised the manuscript. WW analyzed the data and wrote and revised the manuscript.

## Conflict of Interest Statement

The authors declare that the research was conducted in the absence of any commercial or financial relationships that could be construed as a potential conflict of interest.

## Footnotes

1 http://motif-x.med.harvard.edu/motif-x.html

2 http://www.maxquant.org

3 http://string-db.org/

4 http://motif-x.med.harvard.edu/motif-x.html

## Supplementary Material

Figure S1 Phenotyping of *Chlamydomonas reinhardtii* cells treated with 500 nM rapamycin (orange) or with drug vehicle (blue). **(A)** Chlorophyl content per ml, **(B)** Chl content per mg FW, **(C)** Fv/Fm. **(D,E)** Are TLC analysis of total lipid mixtures from *Chlamydomonas* control and rapamycin-treated samples at 0, 24, and 48 h. Lipid mixtures were extracted from and separated on a Silica gel 60 plates using the hexane: ethyl ether: acetic acid (90:7.5:1) solvent composition. Lipid class distribution was visualized under UV light after primuline spraying (Li et al., 2008).

Table S1 Proteins detected and quantified in this work and their abundance changes along two-way ANOVA results, as well as PCA loadings, HCA cluster and functional enrichment analysis for Figure 3.

Table S2 Phosphopeptides detected and quantified in this work and their abundance changes along two-way ANOVA results, as well as PCA loadings.
