# Supplementary figures and images for: Quantitative Phosphoproteomic and System-Level Analysis of TOR Inhibition Unravel Distinct Organellar Acclimation in Chlamydomonas reinhardtii
Source: Front Plant Sci. 2018 Nov 28;9:1590. doi: 10.3389/fpls.2018.01590 (PMC6280106; doi:10.3389/fpls.2018.01590)

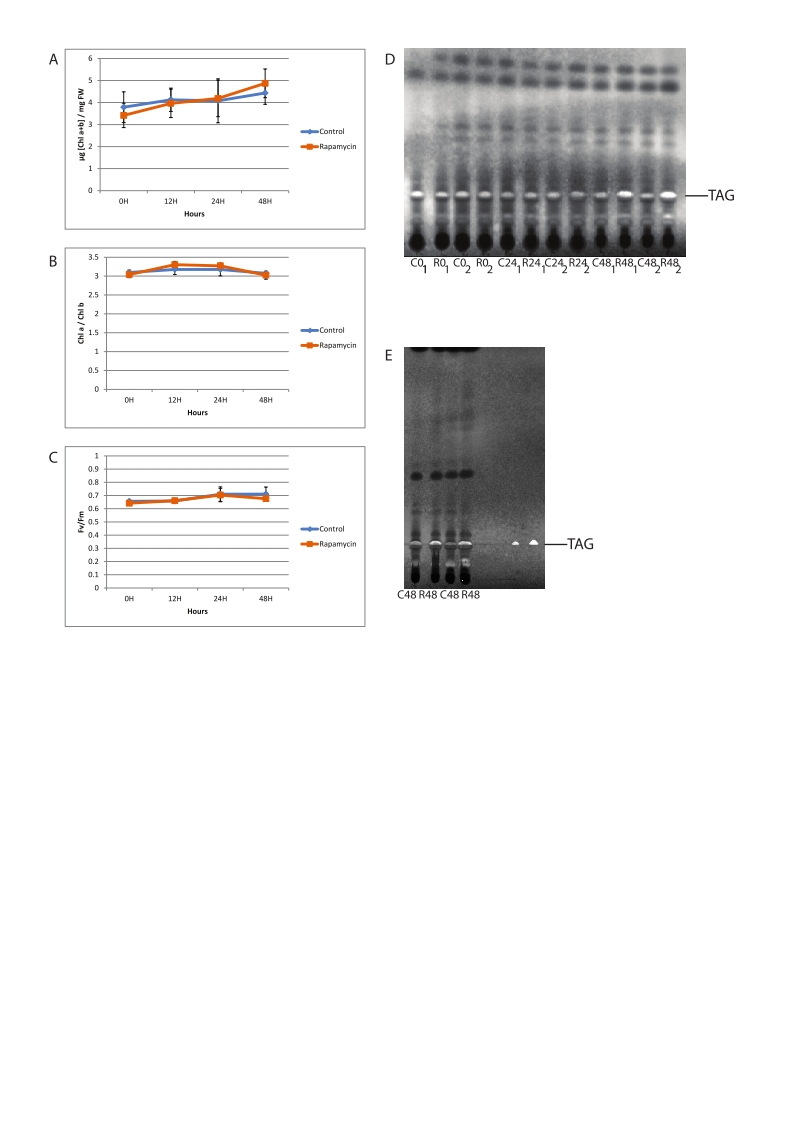

Supplement: Figure S1 — Phenotyping of Chlamydomonas reinhardtii cells treated with 500 nM rapamycin (orange) or with drug vehicle (blue). (A) Chlorophyl content per ml, (B) Chl content per mg FW, (C) Fv/Fm. (D,E) Are TLC analysis of total lipid mixtures from Chlamydomonas control and rapamycin-treated samples at 0, 24, and 48 h. Lipid mixtures were extracted from and separated on a Silica gel 60 plates using the hexane: ethyl ether: acetic acid (90:7.5:1) solvent composition. Lipid class distribution was visualized under UV light after primuline spraying (Li et al., 2008). [file Image_1.JPEG]
